# Supplementary material for: Biomechanical study of anterior and posterior pelvic rings using pedicle screw fixation for Tile C1 pelvic fractures: Finite element analysis
Source: PLoS One. 2022 Aug 25;17(8):e0273351. doi: 10.1371/journal.pone.0273351 (PMC9409507; doi:10.1371/journal.pone.0273351)
Supplement: Attachment — Submitted filename: Response to Reviewers.doc [file pone.0273351.s001.doc]

Dear reviewers,

Thank you so much for your very positive feedback on our study and investing time to provide detailed and insightful comments to improve our paper. Reviewers’ comments were taken carefully and revisions were made accordingly. Edited contents were highlighted in red in the main text. Looking forward to from you soon!

Dear editor,

Thank you so much for your very positive feedback on our study!Our manualscript format has been modified according to PLOS ONE's style requirements.There is no author-generated code in this study.Participant consent was obtained and written consent was signed prior to the start of the study. The type of consent of the participants has been supplemented in the Methods section of the manuscript.This study was supported by Jining Medical University (grant number JYFC2019FKJ186). We have checked the grant number when resubmitting.All relevant data are within the manuscript.We checked author lists and author affiliations in the manuscript to ensure that each author was linked to an affiliation.Ethics Statement outside the Methods section of the manuscript has been removed.Thanks again for your comments on our study.

Respond to reviewer#1

Dear reviewer,

Thank you so much for your very positive feedback on our study！I quite agree with you that this is a calculated model that must first prove itself in clinical practice.We have validated this technique in clinical work with good postoperative results, and this study provides biomechanical evidence for the use of pedicle screws for fixation of anterior and posterior pelvic rings.There are also some literature reports on the clinical application effect of this technology.References are as follows:

Wu XT, Liu ZQ, Fu WQ, Zhao S. Minimally invasive treatment of unstable pelvic ring injuries with modified pedicle screw-rod fixator. J Int Med Res. 2018;46:368–380.

Hua X, Yan SG, Cui Y, Yin Z, Schreiner AJ, Schmidutz F. Minimally invasive internal fixator for unstable pelvic ring injuries with a pedicle screw-rod system: a retrospective study of 23 patients after 13.5 months. Arch Orthop Trauma Surg. 2019;139:489–496.

C. Bi, Q. G. Wang, C. Nagelli, J. H. Wu, Q. Wang and J. D. Wang.Treatment of Unstable Posterior Pelvic Ring Fracture with Pedicle Screw-Rod Fixator Versus Locking Compression Plate: A Comparative Study.Med Sci Monit.2016;22:3764-3770.

We believe that the fixation of the implant in osteoporotic bone can be increased by cement augmentation in older people with FFP-type pelvic injuries.(Schmitz, P., Baumann, F., Acklin, Y.P. et al. Clinical application of a minimally invasive cement-augmentable Schanz screw rod system to treat pelvic ring fractures. International Orthopaedics (SICOT) 43, 697–703 (2019).https://doi.org/10.1007/s00264-018-3988-6.)

Since 2015, a number of biomechanical finite element analysis studies have been published on the treatment of C1 fractures. Nonetheless, we have not found a biomechanical study of the anterior INFIX technique combined with the posterior TIFI technique in the treatment of Tile C pelvic fractures, nor did we retrieve any biomechanical reports on whether the diameter of the pedicle screw affects the fixation effect of unstable pelvic rings.In one biomechanical study of the anterior INFIX technique, better stability occurred with a greater number of screws(Liu L, Fan S, Chen Y, Peng Y, Wen X, Zeng D, et al. Biomechanics of anterior ring internal fixation combined with sacroiliac screw fixation for tile C3 pelvic fractures. Med Sci Monit. 2020;26:e915886). Nonetheless, this study did not compare the biomechanical differences between the two fixation methods of placing screws on the injured and healthy sides of the pubic region.At the same time, the ligaments of the injured side of the pelvis were not removed in this study, and the posterior pelvic ring were fixed with SI screws.Our study supplements the gaps in these previous studies, which is where this study distinguishes the published literature and is also the innovation of this paper.Related description has been stated and added in the introduction section.(page 5-6，line 80-95).

Thanks again for your comments on our study.

Respond to reviewer #2

1. Thank you so much for your very positive feedback on our study！In order to ensure the synchronization between the software and the model, some modifications have been made in the Materials and methods section of this paper, and the modified parts have been marked with red fonts.（page 7-8,line 120-144;page 9,line 155-160;page 11,line 191）
2. Thank you so much for your very positive feedback on our study! We have added the limitations of the current study in the introduction of the present study and marked them in red.（page 5-6,line 81-88）
3. Thank you so much for your very positive feedback on our study！We have added several commonly used methods of stabilizing the posterior pelvic ring and described their use in the Discussion section of the article. Additions have been marked in red font.SI screw fixation is currently a popular posterior fixation method for the treatment of unstable pelvic fractures, but this fixation method has the disadvantages of high technical requirements, repeated fluoroscopy, and high rate of vascular and nerve injury. Anterior SIJ plate fixation is one of the standard methods for the treatment of unstable posterior pelvic ring injuries, but it requires open reduction, and extensive soft tissue dissection can easily lead to complications such as massive pelvic bleeding and infection. Posterior tension band plate fixation achieved functional results consistent with SI screw fixation with reduced intraoperative fluoroscopy time without the risk of nerve or vascular damage associated with SI screw fixation. However, it is sometimes difficult to achieve the correct bending of the plate during the operation. Repeated bending of the plate may reduce its strength and even damage the threads of the threaded holes.（page 18-19,line 327-337）

Thanks again for your comments on our study.
